# Employing a serious game intervention to promote adolescent school children’s perceptions of nursing and midwifery professions

**DOI:** 10.1186/s12912-024-02045-1

**Published:** 2024-06-03

**Authors:** Gary Mitchell, Debbie Rainey, Maria Healy, Tara Anderson, Patrick Stark, Felicity Agwu Kalu, Catherine Monaghan, Mark A Linden

**Affiliations:** School of Nursing and Midwifery, Queen’s University Belfast, Belfast, UK

**Keywords:** Serious game, Intervention, Acceptability testing, Post-primary schools, Education, Career, Nursing, Midwifery

## Abstract

**Aim:**

To test a serious game intervention about nursing and midwifery perceptions among adolescent school children.

**Background:**

Nursing and midwifery professions face challenges in recruitment, with persistent gender stereotypes and a lack of clarity regarding the roles contributing to this issue. This study addresses the need for innovative approaches to reshape perceptions and encourage career exploration in adolescent school children.

**Design:**

Employing a pre/post-test design, this study involved 137 post-primary students aged sixteen or seventeen in the United Kingdom.

**Methods:**

Data collection occurred between November 2022 to April 2023, involving three post-primary schools. Participants engaged with a digital serious game designed to address misconceptions and promote a more accurate understanding of nursing and midwifery. Participants completed pre- and post-intervention questionnaires, including an adapted version of the Nursing as a Career Choice Questionnaire. Statistical analyses included descriptive statistics, paired t-tests, and independent groups t-tests.

**Results:**

The serious game intervention resulted in statistically significant improvements in students’ perceptions across multiple domains. Overall, participants showed higher mean post-test scores (M = 139.57, SD = 15.10) compared to their mean pre-test score (M = 131.06, SD = 14.73) on the questionnaire. The domains of self-efficacy (*p* < .001), job prospects (*p* < .001) and social influences (*p* < .001) exhibited the most substantial positive changes. Female participants showed higher perceptions than males in pre- and post-tests and students from all-girls schools consistently scored higher than those from all-boys and mixed-gender schools.

**Conclusions:**

A serious game about the nursing and midwifery professions appears to be capable of changing perceptions of self-efficacy and job prospects related to nursing and midwifery professions. The study suggests that a serious game intervention may have the potential to change adolescent perceptions of nursing and midwifery professions which may lead to their considering of these when making future career choices.

## Background

The global nursing and midwifery workforce, comprising approximately 27 million individuals, plays a pivotal role in health promotion, disease prevention, and care delivery, constituting nearly 50% of the entire global health workforce [1]. However, the current deficit of registered nurses accounts for greater than 50% of the global shortfall [2], with an estimated 900,000 global shortage of midwives [3]. The World Health Organization [1] estimates that an additional 9 million nurses and midwives are needed by 2030 to meet global demand. Nurses and midwives are integral to healthcare systems, and they have a crucial role in achieving universal health coverage [4, 5]. Further, investing in nurses and midwives proves economically advantageous, as highlighted by the UN High-Level Commission on Health Employment and Economic Growth, showcasing a triple return of improved health outcomes, global health security, and inclusive economic growth [6].

Global shortages in nurses and midwives have led to much research on strategies to promote recruitment and retention in the healthcare workforce [7, 8]. A notable challenge in this regard is the impact of misconceptions from individuals considering enrolment in relevant nursing and midwifery programmes [9–11]. These misperceptions align with those identified in a recent systematic review of the literature which found that while there exists substantial public trust in nursing, it often arises more from admiration for traditional, sentimental stereotypes of selfless, hardworking young women rather than a comprehensive understanding of the profession’s work and impact [12]. A study conducted in the UK showed that around 60% of the public expressed hesitancy toward pursuing a career in nursing [13]. The study, encompassing over 1000 members of the general public from England, identified factors such as ‘handling bodily fluids,’ ‘patient care tasks,’ and ‘job-related stresses’ as the primary deterrents. Furthermore, only 32% of respondents considered leadership a crucial attribute for nursing, while 23% recognised the significance of innovation in the profession. This study therefore demonstrates the public’s lack of understanding of the skilled nature of nursing by revealing widespread hesitancy toward pursuing nursing careers, coupled with a focus on more routine duties and a limited recognition of crucial attributes such as leadership and innovation within the profession [13]. An e-survey also found a public misinterpretation of the role of the midwife and highlighted a lack of understanding of their high level of skill and expertise [14]. These results highlight the prevalence of outdated perceptions about the nature of nursing and midwifery among a considerable portion of the public [15–17].

While there are several interventions that could be employed to promote public understanding of nursing and midwifery, recent research has recommended more contemporary approaches using digital technologies [18–20]. Continuing the exploration of innovative approaches to promote public understanding of nursing and midwifery, gamification and serious games may emerge as a strategy worth investigating in the context of adolescents considering healthcare careers. In recent years, the integration of digital gaming, often referred to as ‘gamification,’ has gained traction in both business and health sectors [21]. Gamification involves applying game-design elements and principles in non-game contexts to engage and motivate users towards specific goals. Within this, ‘serious games,’ designed to fulfil educational needs through interactive gameplay, have become recognised as effective tools in healthcare and education settings [22, 23]. Unlike entertainment-focused games, serious games are specifically designed to address educational objectives by delivering content in an engaging and interactive format [24]. These games often incorporate elements such as challenges, immediate feedback, and progression systems to enhance learning outcomes. While serious games have demonstrated educational benefits for students and professionals in healthcare [25, 26], their application in reshaping adolescent perceptions of nursing and midwifery remains unexplored. Serious games offer a unique opportunity to engage adolescents in meaningful learning experiences while challenging misconceptions and stereotypes associated with nursing and midwifery careers. By immersing players in interactive scenarios that mirror real-world challenges and responsibilities within nursing and midwifery professions, serious games offer an avenue for individuals to gain insights into the complexities and rewards of these careers. Through engaging gameplay experiences, players can explore diverse aspects of healthcare practice, allowing them to develop a deeper understanding and appreciation for the field while fostering interest in pursuing healthcare careers. Therefore, further exploration and research into the potential of serious games as a tool for promoting understanding and interest in nursing and midwifery among adolescents are warranted.

The aim of this study was, therefore, to evaluate the use of a digital serious game among sixteen- and seventeen-year-old students with the goal of addressing and dispelling misconceptions surrounding nursing and midwifery. By engaging young individuals when they are considering their future careers, we aim to present a more accurate understanding of the nursing and midwifery professions. Greater awareness may also potentially influence players to find out more about undertaking a career as a nurse or midwife.

## Methods

### Study design

This research employed a pre/post-test design where each participant acted as their own control. We sought to test whether a brief serious game intervention, lasting approximately three minutes, would be capable of altering post primary student’s perceptions of nursing and midwifery professions. The dependent variable was measured using a modified version of the Nursing as a Career Choice Questionnaire [27]. Our independent variables comprised gender of the students, gender composition of the post-primary school, and pre versus post-test perceptions. Transparent Reporting of Evaluations with Nonrandomised Designs (TREND) reporting guideline informed the reporting of this intervention evaluation. A copy of this is available from the corresponding author on request.

### Participants

Participants were adolescents aged sixteen or seventeen years attending a post-primary school in Northern Ireland. Three post-primary schools were selected to focus on the gender make up of each. Therefore, we included one each of a mixed gender, all-boys and all-girls school. The selection of schools was convenience-based, as they had previously collaborated with the host institution at careers fairs, albeit not specifically focused on nursing or midwifery careers. These schools were situated within a single county in Northern Ireland and, apart from gender, exhibited limited diversity in other demographic aspects.

The chosen sample size allowed for an initial exploration of the intervention’s impact across diverse school settings, prioritising feasibility and potential effectiveness over statistical power calculation in this early-stage study. Therefore, utilising one all-girls school, one all-boys school, and one mixed-gender school allowed for an exploration of the intervention’s impact. Given the study’s nature as an early test of the intervention, the selected sample size provided a reasonable scope for assessing initial effects and gathering preliminary data to inform future research and intervention development. Furthermore, the absence of a power analysis aligns with the typical approach in early-stage intervention studies, where feasibility and potential effectiveness take precedence over statistical power calculation.

### Serious game intervention

The development of a digital serious game, titled “Nursing and Midwifery as a Profession”, (https://games.focusgames.co.uk/QUB_nursing_as_profession/game/) was primarily designed by the authors (GM, DR, MH, FK, CM & ML) in 2022. Initially, the authors consulted with current registered nursing and midwifery students at their university’s monthly ‘student voice forum’ to determine ways in which the professions of nursing and midwifery could be promoted to adolescent school children who could be considering their career options. Several students suggested that the use of a brief intervention would be helpful with a view to dismissing common misconceptions about these professions and promoting potential career pathways. As a result, the authors designed a digital serious game based on student feedback. The web-based serious game operates on any device equipped with an internet connection, utilising HTML5 technology. Players navigate a designated pathway towards the finish line, accomplishing this task by engaging with a series of multiple-choice questions focused on nursing and midwifery. These were drawn from a bank of approximately eighty questions and presented in a random sequence, adding an element of unpredictability to the gameplay.

The intervention was designed alongside guidance from the Northern Ireland Practice and Education Council for Nursing and Midwifery (NIPEC: https://nipec.hscni.net/). NIPEC guidance provided relevant information regarding career pathways, pay scales, course requirements and so on. In the interactive digital serious game focused on the nursing and midwifery career and this was designed for solo play. Upon playing the game, players are presented with a series of questions and their objective is to get as many of these correct within the time limit. Each question challenges players with aspects related to nursing and midwifery careers, such as debunking misconceptions (e.g., determining if nurses and midwives are akin to doctors’ assistants), clarifying work settings (e.g., exploring whether nurses and midwives exclusively work in hospitals), and gauging educational qualifications (e.g., identifying the highest degree qualification achievable – diploma, bachelor, masters, doctorate, etc.). Following each question, players receive informative blurbs that reinforce correct answers or provide insights into the correct response. Players who excel in answering questions accurately can join an online leader board and share their scores, with the view of fostering a sense of achievement and competition.

At the game’s conclusion, players are presented with options to delve deeper into the nursing and midwifery professions. They can access additional resources, including a link to an informative website dedicated to nursing and midwifery careers (https://www.qub.ac.uk/schools/SchoolofNursingandMidwifery/). Moreover, players can opt to watch a 20-minute video offering a more in-depth exploration of careers (https://www.youtube.com/watch?v=Xk9qph4Y8F4) or listen to a forty-five minute podcast featuring current nursing students discussing their experiences and insights about the courses (https://www.youtube.com/watch?v=6RadoRuPRKA). These aspects are not part of the serious game, rather they are placed as an additional information section at the conclusion of the game.

**Figure 1:**
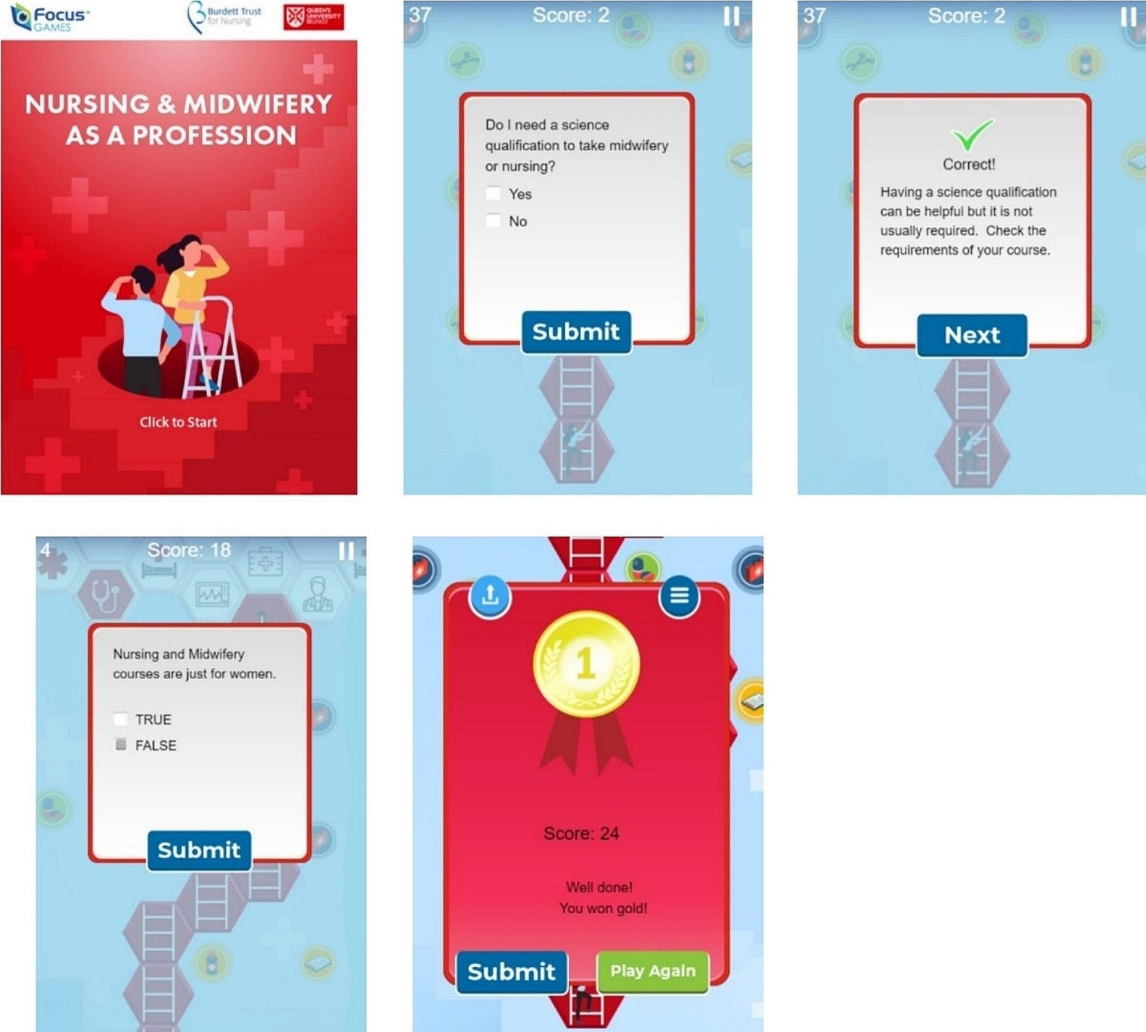
Images of serious game intervention

### Measures

We assessed pre- and post-intervention perceptions of the nursing and midwifery professions using the Nursing as a Career Choice Questionnaire [27]. This questionnaire, known for its well-established psychometric properties, consists of 35 items categorised into six factors. The first factor, personal interest, measured students’ personal interest in the nursing and midwifery professions in relation to, for example, the altruistic and social nature of the job (e.g. ‘Nurses and midwives desire to help others’). Secondly, prior healthcare exposure, explored previous experiences of healthcare which may influence career choice (e.g. ‘In taking care of a sick family member’). Next, the self-efficacy factor, reflected students’ belief about competence to perform actions required of nurses and midwives (e.g. ‘nurses and midwives can make independent decisions at work’). Perceived nature of work related to how the characteristics of the profession were perceived (e.g. ‘nurses and midwives do not mind attending to other’s hygiene needs’). The job prospects factor then focused on future job security (e.g. ‘nursing and midwifery ensures a stable job’). Finally, social influences which may impact career aspirations were measured (e.g. ‘nurses and midwives are well respected’).

To ensure the questionnaire’s relevance to both nursing and midwifery professions, the authors performed an adaptation process. The items were carefully modified to encompass aspects specific to midwifery. For instance, original items like ‘nursing careers reflect well of one’s academic ability’ were adjusted to ‘nursing and midwifery careers reflect well of one’s academic ability.’ This adaptation aimed to maintain the questionnaire’s validity and comprehensiveness in capturing perceptions of both professions. The authors conducted a preliminary face validity assessment by administering the adapted questionnaire to a pilot group of six students aged 16. This small-scale testing aimed to ensure clarity, relevance, and understanding of the questionnaire items among the target age group. While the pilot sample size was limited, the insights gained from this initial assessment confirmed understanding amongst the group.

Participants respond via a 5-point Likert scale from 1 ‘strongly disagree’ to 5 ‘strongly agree’. Scores ranged from 35 to 175. Each subscale had varying potential ranges of scores: 5–25 (personal interest & perceived nature of the job), 6–30 (prior healthcare exposure), 4–20 (self-efficacy), 7–35 (job prospects), 8–40 (social influences). Cronbach’s alpha for the questionnaire had been determined as 0.94. The adapted questionnaire also showed high internal consistency with a Cronbach’s alpha of 0.90. Our post intervention measurement pack also included closed-ended questions intended to assess the acceptability of the serious game to students. Questions included, ‘What did you learn about nursing or midwifery from the game?’, ‘How, if at all, have your views about nursing and midwifery changed from playing the game?’, and ‘Do you think young people of your age in other schools would benefit from playing this game?’

### Ethical approval

This research received full ethical approval from an institutional ethical review board at the lead authors University (Ref: MHLS 22_122). Three teachers, involved with career development at the post-primary schools, acted as gatekeepers for this study. The gatekeepers sent both students and their parents/guardians’ information about the study explaining its purpose along with their rights to confidentiality, benefits or risks of taking part and data protection. Parents/guardians could deny their child’s participation in the study be returning an opt-out form to the gatekeepers. No parent/guardian chose to opt their child out of participation. All participants were aged 16 or older and provided informed consent to participate, collected via an online form, prior to their recruitment. Participants also received a £10 online gift certificate in thanks for their participation. This work was conducted in accordance with the Declaration of Helsinki.

### Data collection

We collected data between November 2022 and April 2023. Having identified three post-primary schools willing to take part in the research, we asked careers teachers (the gatekeepers) to contact students and their parents/guardians on our behalf. Gatekeepers emailed the ethics materials necessary to provide informed consent to both the students and their parents/guardians. Students and their parents/guardians were given a minimum of one week to consider whether they wished to participate in the research. Students were then emailed a link to the pre-test questionnaire pack which contained consent questions, together with some basic demographic information (gender, name of school and experience of the nursing profession) and the adapted Nursing as a Career Choice Questionnaire. Approximately two weeks later students were emailed a further link to the serious game. After playing this, they were asked to complete the post-test questionnaire. Students provided their email address to enable matching between their pre and post-tests questionnaires. After matching had taken place, all email addresses were deleted from the dataset.

### Data analysis

The pre- and post-test datasets were matched prior to analysis using email addresses. All analyses were conducted in SPSS version 28. The nursing and midwifery perceptions questionnaire was coded on a Likert scale ranging from 1 (strongly disagree) to 5 (strongly agree) with higher scores reflecting more positive perceptions. Total and mean scores were calculated for each domain. Descriptive statistics were first performed to find details of gender distribution, age, and school type (all girls, all boys and mixed gender). Box plots were drawn for differences in pre- and post-test scores for each domain of the questionnaire to examine the data for outliers. Tests of normality were also performed on these variables. Paired t-tests were performed on each domain of the nursing and midwifery perceptions questionnaire to test the null hypotheses that the mean difference in scores in each domain between the pre- and post-test conditions will be equal to zero. A total score for both the pre- and the post-questionnaire was calculated, and an independent t-test was also conducted to investigate gender differences on perceptions of nursing and midwifery.

In total, nine comparison analyses were conducted in this study. Therefore, a Bonferroni correction was applied to the alpha value when determining the statistical significance of the results of these analyses to reduce the risk of false positives associated with multiple comparisons [28]. Alpha (0.05) was divided by this total number of comparisons (9) to give an alpha value of α = 0.0056. Results were therefore only considered to be statistically significant if their associated *p*-value was 0.0056 or below.

## Results

In total, 137 participants (Table 1) were recruited to evaluate the impact of a serious game on perceptions of nursing and midwifery professions as assessed by pre- and post-questionnaires.

**Table 1:** Participant descriptive statistics

		*N*	%
Gender	Female	73	53.3%
	Male	58	42.3%
	Non-Binary	1	0.7%
	Transgender	1	0.7%
	Other	1	0.7%
	Missing	3	2.2%
Age	16	123	89.5%
	17	9	6.6%
	Missing	5	3.6%
School	All-Girls	58	42.3%
	All-Boys	47	34.3%
	Mixed-Gender	29	21.2%
	Missing	3	2.2%

### Missing data

A total number of 137 participants responded to at least one of the questionnaire time-points in the study, i.e. pre-test, post-test, or both time-points. 134 participants completed pre-testing while 137 participants completed post-testing. If a participant was missing either pre-test or post-test data, they were excluded from paired analysis.

### Closed-ended questions

Prior to playing the game, 25.4% (*n* = 34) stated that they were considering a career in nursing or midwifery, while 66.4% (*n* = 89) stated they were not considering this career, and 8.2% (*n* = 11) stated they might consider it. Post playing the game, 37.2% (*n* = 51) stated that they would consider a career in nursing or midwifery, while 51.8% (*n* = 71) stated they would not consider this career, and 10.9% (*n* = 15) expressed uncertainty or mentioned they might consider it. In response to the question, ‘Do you think young people of your age in other schools would benefit from playing the game?’, 81.8% (*n* = 112) stated yes, 13.1% (*n* = 18) stated maybe and 5.1% (*n* = 7) stated no.

### Pre and post intervention changes in perceptions

Paired t-tests were conducted on overall questionnaire pre- and post-test scores, as well as on each domain of the questionnaire. Participants showed higher mean post-test scores (*M* = 139.57, *SD* = 15.10) compared to their mean pre-test score (*M* = 131.06, *SD* = 14.73) on the overall questionnaire, a statistically significant mean increase of, *M* = 8.51, 95% CI [6.01, 11.02], *t*(133) = 6.73, *p* < .001, with medium effect size (*d* = 0.58).

Paired t-tests on each domain of the questionnaire showed that the intervention increased student perceptions about nursing and midwifery professions across all six domains of the questionnaire. For three domains (self-efficacy, job prospects, and social influences), these differences were statistically significant at the *p* < .001, i.e., below the Bonferroni-corrected alpha cut-off of *p* = .0056. The difference between pre- and post-test means for personal interest (*p* = .05), prior healthcare exposure (*p* = .06), and perceived nature of work (*p* = .03) were not statistically significant. Table 2 shows the means, standard deviations, 95% CIs and effect sizes for these tests.

**Table 2:** Results of paired t-tests

Scale	Pre Mean (SD)	Post Mean (SD)	95% Cis & *p* values	Cohen’s d
Overall Questionnaire	131.06 (14.73)	139.57 (15.10)	6.01 to 11.02 (*p* < .001)*	0.58
Personal interest	21.92 (2.61)	22.28 (2.60)	-0.06 to 0.79 (*p* = .09)	0.15
Prior healthcare exposure	22.60 (3.51)	23.10 (3.48)	-0.11 to 1.10 (*p* = .11)	0.14
Self-efficacy	14.16 (2.70)	15.94 (2.92)	1.21to 2.34 (*p* < .001)*	0.53
Perceived nature of work	20.77 (2.91)	21.23 (2.44)	-0.31 to 0.96 (*p* = .07)	0.16
Job prospects	24.08 (4.53)	27.17 (4.29)	2.30 to 3.89 (*p* < .001)*	0.67
Social influences	27.52 (4.50)	29.85 (4.76)	1.41 to 3.25 (*p* < .001)*	0.43

*Statistically significant

### Gender differences in student perceptions

Independent t-tests were conducted to explore gender differences in student perceptions of the professions at both the pre- and post-test level on total questionnaire scores. Female participants scored statistically significantly higher than male participants at both time-points. Further details are presented in Table 3.

**Table 3:** Results of independent t-tests

Time point	Female Mean (SD)	Male Mean (SD)	95% Cis & *p* values	Cohen’s d
Pre-test	134.47 (12.31)	126.86 (16.78)	2.57 to 12.64 (*p* = .003)*	0.53
Post-test	143.71 (12.94)	133.86 (15.86)	4.87 to 14.83 (*p* < .001)*	0.69

*Statistically significant

### Impact of school type on student perceptions

Participants attending an all-girls school scored consistently higher across all domains of the questionnaire at both pre- and post-test levels than both those attending an all-boys school and a mixed gender school. See Table 4 for means and standard deviations (SD) for pre- and post-perceptions scores by school type. These results are also presented based on gender for the mixed-gender school to highlight any differences which may be due to gender rather than school type. Participants attending an all-girls school also scored higher than female participants who attended the mixed-gender school.

**Table 4:** School type descriptive statistics

		All-Girls Mean (SD) *N* = 58	All-Boys Mean (SD) *N* = 47	Mixed-Gender Mean (SD)		
				Total (*N* = 29)	Female (*N* = 18)	Male (*N* = 11)
Personal Interest	Pre	23.09 (2.10)	20.70 (2.58)	21.55 (2.64)	21.83 (3.03)	21.09 (1.87)
	Post	23.19 (1.84)	21.64 (2.40)	21.52 (3.57)	22.33 (2.77)	20.18 (4.42)
Prior Healthcare Exposure	Pre	23.91 (3.56)	21.32 (3.38)	22.07 (2.72)	22.06 (2.96)	22.09 (2.43)
	Post	24.02 (3.67)	22.06 (3.35)	22.93 (2.84)	22.56 (3.35)	23.55 (1.69)
Self-Efficacy	Pre	14.69 (2.36)	13.66 (2.86)	13.93 (2.95)	13.50 (3.37)	14.64 (2.06)
	Post	17.05 (2.62)	14.68 (2.85)	15.76 (2.81)	15.94 (3.26)	15.45 (1.97)
Perceived Nature of Work	Pre	21.86 (2.59)	19.85 (3.16)	20.07 (2.39)	19.94 (2.44)	20.27 (2.41)
	Post	22.17 (1.96)	20.51 (2.71)	20.52 (2.28)	20.44 (2.45)	20.64 (2.06)
Job Prospects	Pre	24.50 (3.67)	23.04 (5.19)	24.93 (4.77)	25.06 (5.08)	24.73 (4.45)
	Post	28.53 (3.41)	25.62 (4.57)	26.97 (4.63)	26.72 (4.68)	27.36 (4.76)
Social Influences	Pre	27.74 (4.53)	27.79 (4.45)	26.66 (3.84)	25.89 (4.03)	27.91 (3.30)
	Post	30.95 (4.47)	28.98 (5.05)	29.07 (4.54)	29.00 (4.41)	29.18 (4.96)

## Discussion

This study introduces a novel intervention, the digital serious game titled “Nursing and Midwifery as a Profession,” designed to address misconceptions and promote a more accurate understanding of nursing and midwifery among 16-17-year-old students. The results demonstrate positive changes in students’ perceptions across the domains of self-efficacy, job prospects and social influences, indicating that the serious game intervention did impact on how participants viewed the nursing and midwifery professions. The findings align with previous research on interventions aimed at altering perceptions of healthcare professions, emphasising the potential of innovative approaches like serious games in reshaping attitudes [29–31].

The serious game elicited statistically significant increases in perceptions across three domains of the questionnaire: self-efficacy, job prospects and social influences. On the other hand, three domains did not show statistically significant increases: personal interest, previous healthcare exposure, and perceived nature of the work. By engaging students in a serious game that showcased the skills, challenges, and rewards of nursing and midwifery, the game may have boosted student confidence in their ability to pursue such careers (self-efficacy), provided insight into the potential career paths and opportunities available in these professions (job prospects), and highlighted the positive societal impact and support networks associated with nursing and midwifery (social influences). The significant increases in perceptions of self-efficacy, job prospects, and social influences resulting from the serious game intervention likely stem from its interactive nature, immediate feedback system, and focus on myth-busting and career exploration within nursing and midwifery. By engaging students in a digital format featuring multiple-choice questions and explanatory blurbs, the game educated participants about the skills, challenges, and rewards associated with these professions. Moreover, the multiple-choice questions (MCQs) likely played a crucial role in influencing the significant outcomes, as they were tailored to focus more on areas such as myth-busting, career prospects, and societal impact, which aligned with the domains showing improvement, such as self-efficacy, job prospects, and social influences. This targeted approach may have contributed to reinforcing positive perceptions and attitudes towards nursing and midwifery careers among participants.

Conversely, the domains of personal interest, previous healthcare exposure, and perceived nature of the work may not have shown statistically significant increases due to various factors. Personal interest in a career is often influenced by individual preferences and experiences that may not be easily altered by a single intervention like a serious game. Similarly, participants’ previous exposure to healthcare settings or professions might have already shaped their perceptions, making it more challenging for the intervention to create significant changes in this regard. Additionally, altering deeply ingrained perceptions of the nature of nursing and midwifery work may require more long-term interventions beyond the scope of a single serious game session. This could also be attributed to the limitations of the game format in addressing deeply ingrained perceptions, individual preferences, and experiences. For instance, altering perceptions of the nature of nursing and midwifery work, which may be based on deep-rooted stereotypes, might require more extensive interventions beyond the scope of a single game session. Similarly, participants’ previous exposure to healthcare settings or professions might have already shaped their perceptions, making it challenging for the game to create significant changes in this regard. Therefore, while the serious game intervention showed promising results in certain domains, its effectiveness in altering deeply entrenched perceptions or preferences may be limited.

With regards to gender of participants, the comparison of male and female student perceptions before and after engaging with the serious game revealed females to score statistically significantly higher at both levels. This highlights the traditional gender-related barriers to nursing and midwifery careers that have been well documented [32, 33]. Although it is promising that both male and female perceptions increased following engagement with the game, females continued to score higher suggesting males may need further education regarding nursing and midwifery careers beyond that of a short serious game. It is important that gender-based disparities in post-primary school students’ perceptions are addressed to help dispel stereotypes and encourage a diverse pool of individuals to explore becoming nurses or midwives [34].

The reason for these results may be because the field of caregiving, particularly within the healthcare sector, is heavily associated with femininity, and both the nursing and midwifery professions have a solid foundation rooted in female representation [35, 36]. Despite concerted efforts to achieve greater gender equity seen in many other professions, the representation of men in nursing remains around 10% in high-income countries [37, 38], with approximately 2.5% of men in midwifery [39]. Various factors contribute to this persistent gender imbalance. The media often portray men in nursing negatively, perpetuating stereotypes that hinder a more diverse representation [40] and male midwives are often from these platforms. Discrimination against male nurses has also been reported to persist among patients and staff [41–44]. Similarly, the bias against male midwives from their female midwifery colleagues is highlighted with some expressing how they believed in diversity, while others had concerns about men in the profession [39]. Historically, a range of stereotypical believes exist continuing this bias and hindering men becoming midwives; for example, that women prefer a female midwife or that male midwives cannot care effectively for women because they cannot become pregnant and give birth [36]. Research indicates that the low participation of men in nursing can be traced back to hesitancy among career teachers to recommend nursing as a viable career option for male individuals [45, 46].

Furthermore, the perceived low pay and status of nursing and midwifery as professions may have detrimental effects on boys’ perceptions [47]. Boys might view nursing as an extension of women’s work with low social status, fostering the belief that nursing is not a suitable profession for men [48, 49]. The stigma and negative perceptions associated with nursing and midwifery as low-status jobs could reinforce boys’ reluctance to consider nursing as a viable career option. Additionally, stereotypes, prejudices, and the relatively lower remuneration in the healthcare sector compared to other professions may further dissuade boys from pursuing nursing [50]. These factors collectively create barriers for boys in considering nursing as a career choice, highlighting the urgent need for a shift in societal attitudes and the establishment of a male-inclusive environment to encourage greater participation of boys in nursing and midwifery professions [47–50]. This context may contribute to the observed lower baseline and maximum perceptions among boys in this study.

Interventions like the serious game, may go some way to help challenge and dispel the perception that nursing, and midwifery are exclusively a profession for women, recognising its inclusivity and diverse appeal. Although the serious game intervention exhibited positive effects across three of the six above named domains, distinctions based on school type were also noted, with students from the all-girls school consistently scoring higher across all domains. This effect of school type was seen in addition to gender differences as those attending an all-girls school also scored higher than females who attended a mixed-gender school. This observation concords with another study which employed the ‘Nursing as a Career Choice Questionnaire’ [27] to assess their ‘Make a Difference with Nursing’ intervention among post-primary school students [29]. While the precise reason for this discrepancy remained unclear, the authors suggested that career’s teachers at an all-girls school may be more likely to discuss nursing and midwifery careers with their students, possibly due to the traditionally female dominated nature associated with nursing careers [35]. It is important, therefore, that interventions address such influences and increase the promotion of nursing and midwifery careers within mixed-gender and all-boys schools. Our findings highlight the importance of considering environmental and contextual factors such as school type and information provided by careers teachers in designing interventions and tailoring approaches to address specific concerns or preferences within diverse educational settings.

The effectiveness of the serious game in positively influencing perceptions of nursing and midwifery professions can be attributed to its alignment with the pedagogical principles of constructivism [51, 52]. By incorporating interactive questions and myths within the serious game, participants were engaged in active learning experiences, allowing them to construct their own understanding of nursing and midwifery concepts. The asynchronous nature of the gameplay also facilitated self-directed learning, enabling users to progress at their own pace and engage with feedback mechanisms that reinforced correct answers. Moreover, the incorporation of game-based learning principles, such as challenge, feedback, and interactivity, may have further enhanced user engagement and motivation as noted in previous studies [53, 54]. This aligns with previous research demonstrating the effectiveness of serious games in healthcare education, highlighting their potential to foster motivation and knowledge acquisition among learners. Furthermore, in the context of nursing and midwifery, where predetermined gender biases or prejudices may exist, the self-led nature of the multiple-choice question format allows players to interpret and shape their learning experiences based on their unique backgrounds and perspectives. This aspect of individual interpretation and knowledge construction is fundamental to the constructivist approach, as it recognises the diversity of learners and their ability to create personal meaning from educational content. Therefore, this study’s serious game, focused on dispelling misconceptions about nursing and midwifery, adds to the growing body of literature supporting the efficacy of gamification in healthcare education [22, 55, 56]. However, given the scope of this study, the authors also acknowledge the need for ongoing evaluation, considering factors such as entertainment value, usability, and learner attitudes, to ensure the effectiveness and relevance of this serious game among adolescent school children.

### Strengths and limitations

The study has several notable strengths that enhance the reliability and relevance of its findings. Firstly, the use of a well-established questionnaire, even with minor modifications, provides a structured and standardised approach to measuring perceptions of the nursing and midwifery professions. The employment of a pre- and post-test design allows for an examination of the intervention’s impact before and after playing. Moreover, the inclusion of diverse school types, including all-girls, all-boys, and mixed gender schools, contributes to the generalisability of the study’s findings across various educational settings. Additionally, the explicit consideration of both male and female student perceptions ensures a deeper understanding of the intervention’s effects across genders. Overall, these methodological choices strengthen the study’s internal and external validity, providing a robust foundation for the interpretation of results and potential implications for future nursing and midwifery recruitment interventions.

While our study had several strengths, there were also some limitations. Firstly, the questionnaire used in our study underwent important modifications, including the addition of items relevant to midwifery, and was not formally revalidated. Nevertheless, we prioritised face validity to ensure the questionnaire’s relevance and appropriateness for the study context. The addition of midwifery to the questionnaire also meant that participants could not distinguish their perceptions of nursing and midwifery separately. This may have impacted findings as participants could not express any difference in perceptions between the two careers; for example, if one was more highly regarded as a career choice than the other the inability to separate this may have resulted in lower scores for the professions taken together.

Secondly, our study employed a self-report measure to assess perceptions of the nursing and midwifery professions. While we acknowledge there is a susceptibility to social desirability bias, efforts were made to mitigate this by ensuring students had no direct contact with the research team. Thirdly, our sample size was small and localised to three schools within one region of the UK, therefore caution is warranted in generalising the results. Furthermore, it is essential to note that our intervention focused on specific nursing and midwifery degree programmes offered in the UK (England, Scotland, Wales and Northern Ireland), raising questions about the broader applicability of our findings. In addition, it is not possible to draw conclusions regarding the retention of any changes in perceptions as post-test data was obtained directly after engagement with the game. An additional questionnaire at a later time-point may help to explore any maintained impact of the game.

## Conclusions

This study contributes valuable insights into the use of a digital serious game to alter perceptions of nursing and midwifery among adolescents. The positive changes observed in three out of six domains, the lack of significant gender-based differences, and the consideration of school type variations provide a comprehensive understanding of the intervention’s impact. The findings emphasise the potential of innovative approaches, like serious games, to influence attitudes and contribute to the broader goal of addressing workforce shortages and promoting diverse participation in nursing and midwifery. Future research could explore additional dimensions, including long-term effects and the scalability of such interventions across different cultural and educational contexts.

## Data Availability

The datasets used and/or analysed during the current study are available from the corresponding author on reasonable request.
